# Sex allocation is color morph-specific and associated with fledging condition in a wild bird

**DOI:** 10.1093/beheco/arae039

**Published:** 2024-05-17

**Authors:** Amandine Tooth, Chiara Morosinotto, Patrik Karell

**Affiliations:** Department of Biology, Lund University, Sölvegatan 37 (Ecology Building), SE-223 62 Lund, Sweden; Department of Biology, Lund University, Sölvegatan 37 (Ecology Building), SE-223 62 Lund, Sweden; Bioeconomy Research Team, Novia University of Applied Sciences, Raseborgsvägen 9, FI-10600 Raseborg, Finland; Department of Biology, University of Padova, Via U. Bassi 58/B, 35131 Padova, Italy; National Biodiversity Future Center (NBFC), Piazza Marina 61, I-90133 Palermo, Italy; Department of Biology, Lund University, Sölvegatan 37 (Ecology Building), SE-223 62 Lund, Sweden; Bioeconomy Research Team, Novia University of Applied Sciences, Raseborgsvägen 9, FI-10600 Raseborg, Finland; Evolutionary Biology Center, Department of Ecology and Genetics, Uppsala University, Norbyvägen 18D, SE-752 36 Uppsala, Sweden

**Keywords:** early life condition, fitness, life history strategy, melanism, reproductive trade-off, sex ratio, genetic polymorphism

## Abstract

Melanin-based color polymorphism is predicted to evolve and maintain through differential fitness of morphs in different environments, and several empirical studies indicate that life history strategies, physiology, and behavior vary among color morphs. Sex allocation theory predicts that parents should adjust their sex allocation based on differential costs of raising sons and daughters, and therefore, color morphs are expected to modify their brood sex ratio decisions. In color polymorphic tawny owls (*Strix aluco*), the pheomelanistic brown morph is associated with higher energy requirements, faster growth, and higher parental effort than the gray morph. As hypothesized, we find that brown tawny owl mothers produced more daughters in early broods and more males in late broods, whereas gray mothers did the opposite. At fledging, daughters of early broods and of brown mothers were heavier than those of late broods or gray mothers. Hence, larger and more costly daughters appeared to benefit more than males from being born to brown mothers early in the season. Brown mothers breeding later in the season produced more cheap sons, while gray mothers face fewer challenges under limited resources and favor daughters. These findings suggest that environmental conditions influence brood sex allocation strategies of genetically determined color morphs differently.

## Introduction

Early sex allocation theory sought to explain equal ratios of male and female offspring observed in many taxa, with an understanding that resources are divided between the production of sons and daughters. A key model in evolutionary biology, Fisher’s principle assumes equal costs of producing males and females; the initially underrepresented sex has higher fitness returns than the common sex because it faces less competition for mates, and therefore, the production of the lesser sex is favored by selection (Fisher 1930). A population under this frequency-dependent selection is expected to have an equal sex ratio (50:50) or fluctuate closely around this value. However, the development of sex allocation theory has highlighted various instances of unequal sex ratios (Hamilton 1967; Charnov 1982; Komdeur et al. 1997; West and Sheldon 2002; Shuker and West 2004; Alonso-Alvarez 2006; Donald 2007; Cockburn et al. 2009). Thus, assumptions of Fisher’s principle can be violated in cases and contexts when fitness returns vary depending on the sex of the offspring.

The Trivers-Willard hypothesis predicts that parents may skew their offspring ratios in favor of one sex or the other (sex ratio adjustment) based on parental condition and offspring cost, such that the adjustment improves reproductive success (Trivers and Willard 1973). However, while clear examples of sex ratio adjustment have been described in relation to, for example, recruitment (Dijkstra et al. 1990), parental investment (Ellegren et al. 1996), or mate attractiveness (Sheldon et al. 1999), the heritable components of variation in adjustment mechanisms are poorly understood (Hasselquist and Kempenaers 2002). In vertebrates with chromosomal sex determination, there has also been a debate over whether the plethora of studies demonstrating sex ratio biases (Hardy 2009; Bowers et al. 2015) are simply small scale variation around the expected 50:50 sex ratios predicted by Fisher’s model (Fisher 1930), because of the constraints inherent in the sex determination mechanisms (Charnov 1982). However, in a meta-analysis West and Sheldon (2002) concluded that the inherent sex determination mechanisms do not necessarily constrain facultative sex ratio adjustment but that environmental predictability can be a major determinant of the adaptive nature of sex ratio adjustment. Hence, constraints in resource availability are expected to affect sex allocation (West and Sheldon 2002). Accordingly, experimental egg removal studies of sexually size dimorphic birds provide strong evidence that individual food resources affect sex allocation decisions. Under constrained food conditions, both lesser black-backed gulls *Larus fuscus*, and great skuas *Catharacta skua* (Kalmbach et al. 2001), overproduce the smaller sex.

A powerful model to evaluate intraspecific variation in sex allocation decisions due to resource sensitivity is color polymorphism. This is because color morphs are expected to be adaptations to different environmental conditions (Ford 1945). In vertebrates, melanin-based color polymorphism has been found to be associated with differences between morphs in physiology and behavior that can impact reproductive strategies and life history trade-offs (Roulin 2004, 2014), which are expected to lead to differences in life history strategies. In an experimental study of color polymorphic tawny owls, Emaresi et al. (2014) found that feeding investment in offspring by dark pheomelanic brown males is consistent and independent of environmental conditions, whereas pale gray males adjust their reproductive effort relative to environmental variability. In accordance, brown tawny owl parents consistently produce fledglings in better condition than gray or mixed pairs (Morosinotto et al. 2020). Moreover, Morosinotto et al. (2020) also show that there is a seasonal decline in offspring condition (offspring in late broods are in worse condition at fledging), except when they are raised by parents of the brown morph. Since tawny owls display reverse sexual dimorphism, where females are 15% to 25% larger than males (Sunde et al. 2003), we can expect substantial variation in fitness return from sons and daughters (see also McDonald et al. 2005) depending on parental color morph and seasonal timing.

In this paper, we use the same tawny owl model system and predict differences in offspring sex allocation strategies between the gray and brown color morphs of the tawny owl. We measure offspring sex ratios and sex-specific offspring conditions in a Finnish population of tawny owls over a 10-yr period in relation to the timing of breeding and parental color polymorphism. The tawny owl shows a pronounced seasonal decline in both clutch size and offspring mass at fledging (Kekkonen et al. 2008; Morosinotto et al. 2020), which indicates that parents breeding late in the season are more resource limited than early ones. Due to the reverse size dimorphism observed in this species, we predict male-biased sex ratios and reduced condition of female offspring in resource-limited late broods. Moreover, offspring of brown mothers have been found to be more sensitive to poor food conditions but grow better than offspring of gray mothers under ad lib conditions (Piault et al. 2009), and offspring of brown parents fledge at greater mass than offspring of gray parents (Morosinotto et al. 2020). Therefore, we predict that brown mothers produce broods that are more strongly male-biased in late broods and more female-biased in early broods compared to gray mothers. Daughters are expected to benefit more from being raised under favorable food conditions compared to sons (Brommer et al. 2003), and this effect is expected to be stronger in broods of brown mothers (and reinforced if the father is also brown) if individuals of the brown morph consistently provide higher quality care (Emaresi et al. 2014).

## Material and methods

The tawny owl (*Strix aluco*) is a common nocturnal, territorial forest-dwelling bird of prey in temperate Europe. A long-term monitoring program of tawny owls has been conducted on an annual basis since 1979 in western Uusimaa, Southern Finland (60°15ʹ N, 24° 15ʹ E), and we used blood samples and life history data collected in 2009 to 2019 for this study. The study area, ~500 km^2^ in size, includes approximately 200 nest boxes available for tawny owl breeding across agricultural and forested landscapes.

To collect information on the breeding biology of tawny owls, nest boxes were visited several times during the annual breeding season. In early to mid-April, nest boxes were regularly checked for eggs, hatching and to record brood size. Offspring wing length was used to determine owlet age, and subsequently, hatching date and laying date, based on a standard growth curve. During the nestling period, both parents were trapped in individual nest boxes, ringed, aged based on molting patterns (Karell et al. 2009, 2013), and sexed (only females have a brood patch during breeding). Additionally, wing length and body mass were measured to assess parental condition, and adult plumage color was scored. Plumage color was assessed using a point system based on pheomelanin (red) pigmentation in 4 different parts of the birds’ plumage: facial disk, back, breast, and general appearance, according to Brommer et al. (2005). This resulted in a score from 4 (a pale gray-colored individual virtually without brown pigments) to 14 (a reddish-brown individual) used to categorize each individual as being of either the gray morph or brown morph. Finally, the nest boxes were revisited when offspring were estimated to be 25 to 28 d old, right before fledging, to ring offspring, collect blood samples for molecular sexing (stored in ethanol or SET buffer, at −20 °C until time of analysis), and record offspring body mass, wing length, and color morph (either gray or brown, see details in Morosinotto et al. 2020).

### Molecular sexing

A total of 369 blood samples of tawny offspring were collected during the 2009 to 2019 breeding seasons as part of the long-term monitoring program and stored in ethanol (2009, 2011, 2014, 2016 to 2018), in SET buffer (2019) or directly as red blood cells in −80 °C (2013, 2015) were used to determine sex of individual offspring (Molecular Ecology and Evolution Lab, Lund University, Lund, Sweden) and provide data on offspring sex ratios for sampled broods. There were no samples collected for this analysis in 2010 and 2012.

DNA was extracted during spring 2019 from the blood samples using an ammonium acetate (NH_4_Ac) protocol for avian blood (adapted from Nicholls et al. 2000; see methods in Morosinotto et al. 2021). Briefly, a small piece of dried blood was collected and placed in a tube with 125 μl of SET buffer; for samples collected in 2019, 125 μl of SET buffer and sample were selected. To all the samples, SDS and Proteinase K were added and then digested overnight at 56 °C. 125 μl of NH_4_Ac were then added, followed by incubation for 60 min at room temperature. The samples were then centrifuged for 15 min at 13,000 rpm, the supernatant was collected, and 500 μl of ice-cold 95% ethanol was added to it. The samples were centrifuged again with same time and speed, and 250 μl of ice-cold 70% ethanol was added and removed. The resulting DNA pellets were then left overnight to air dry, 50 μl of TE buffer was added, and the samples were left for a few days at 4 °C to dissolve the pellet. DNA samples were quantified with an accuracy of ± 10 ng/µl using a NanoDrop™ 2000 Spectrophotometer (Thermo Fisher Scientific, Waltham, MA) and diluted using ddH_2_O from their initial concentrations to 25 ng/µl.

All the samples were sexed using PCR-based methods (using a modified protocol from Kekkonen et al. 2008; see details in Morosinotto et al. 2021) using two primers (2550F 5ʹ-GTTACTGATTCGTCTACGAGA-3ʹ and 2718R 5ʹ-ATTGAAATGATCCAGTGCTTG-3ʹ) specific to the CHD gene (Griffiths et al. 1998). Briefly, the PCR mix included 1 µl of DNA, 0.1 µl AmpliTaq ® DNA Polymerase (5U/µl), 2.5 µl PCR Buffer, 1.5 µl 25 mM MgCl_2_ (all: Applied Biosystems, Foster City, CA), 2.5 µl 1 mM dNTPs (Thermo Fisher Scientific), 1 µl of each primer (10 µM), 3 µl Bovine Serum Albumin (1.00 mg/ml; Invitrogen, Carlsbad, USA) and then ddH_2_O to reach a total volume of 25 µl. Four wells in each PCR plate contained DNA of parent controls of known sex (2 males and 2 females) to determine successful amplification. The analysis was performed in a GeneAMP® PCR System 9700 thermal cycler (Applied Biosystems) using a modified protocol from Fridolfsson and Ellegren (1999): 94 °C for 2 min, 10 cycles at 94 °C for 30 s, 30 s at 60 °C lowered one-half degree per cycle, and 1 min at 72 °C, followed by 30 more cycles at 94 °C for 30 s, 30 s at 50 °C and 1 min at 72 °C and a final 10 min extension at 72 °C. Following amplification, the PCR products were separated by gel electrophoresis on 2% agarose gels stained with GelRed®, using 3.5 µl of PCR product and 3.0 µl loading dye per well. A single band around 650 bp corresponds to ZZ (male), and this first band with an additional band around 2,000 bp corresponds to ZW (female) (Griffiths et al. 1998). Of the 369 samples on which DNA extraction, PCR amplification, and gel electrophoresis were performed, 366 were successfully sexed.

### Statistical analyses

Brood-level offspring sex ratio data were fit to a generalized linear mixed-effects model (glmer, binomial distribution) to test for relationships between parental characteristics (condition, color morph) and offspring brood sex ratio in complete broods only (*n* = 79 broods where all the surviving nestlings could be sampled and sexed). A binomial variable “brood sex ratio,” was generated in R by combining variables on the number of females and the number of males sexed using the cbind() function. We ran 3 comparable generalized linear mixed models (glmer) with binomial errors to test if the sex ratio depended on the parental color morph. In the first model, we used laying date and parental “pair morph” (mother morph × father morph: Gray × Gray, G × Brown, BxG, or BxB) as fixed factors. Female mass was also included as a fixed factor to see the effect of maternal condition on offspring sex ratio. “Female ID” (unique, individual female ring number) and “year” were included as random factors to account for annual variation and to avoid pseudoreplication of broods with the same mother. Interactions between laying date and parental morph were included to test any morph-specific effects of timing of breeding on brood sex ratio. Two additional and otherwise identical models were run where “pairmorph” was replaced with “mother morph” (either brown or gray) to highlight the putative effects of the color morph of the mother. We also ran a similar model with “father morph” as fixed effect, but it did not show any statistical or biologically relevant pattern, and thus results for that model are not shown.

It is common in tawny owls that not all eggs hatch, which affects the sex ratio of the brood. Therefore, our estimates of brood sex ratios in the full data set (*N* = 79 broods) are shortly prior to fledging, and we cannot exclude that differential mortality of eggs are the primary cause of sex ratio bias. To make a more explicit test of sex ratio variation at egg laying, we therefore ran an identical model but on a subset of the data (*N* = 32 broods) where only broods in which all laid eggs which survived to fledging (and all offspring were successfully sexed) were included.

Offspring condition data, as measured by (standardized) offspring mass, were fit to two separate linear mixed effects models (lmer) that tested how the individual morph of the offspring, and the mother, respectively, would affect offspring condition. On an individual level, the maternal morph was included in the model to consider any effect related to the reproductive behavior of either morphs, such as maternal effects on egg quality or parental provisioning (Emaresi et al. 2014). “Year” and ‘Brood ID’ (unique ID of a certain brood in a certain year) were factors included as random effects to account for annual environmental variation and non-independence between siblings. Laying date was included in the model, and the interactions between morph (parental or offspring) and offspring sex, as well as between laying date and offspring sex or pair/maternal morph, were also considered to evaluate sex-specific effects of laying date or morph on condition. Also, the offspring condition model was run with paternal morph as an explanatory variable (replacing offspring morph/maternal morph), but the model did not show any statistical or biologically relevant pattern related to paternal morph, and thus, results for that model are not shown. The variables (body) mass, wing (length) were standardized to zero mean ± 1 SD. All statistical analyses were performed using the statistical program R version 4.2.1 (R Core Team 2022) and the *lme4* package (Bates et al. 2015).

All animal sampling was approved by the ethical board for animal experiments (ESLH-2009-01489-YM-23, ESAVI-1592/04.10.03/2011, ESAVI/2195/04.10.07/2014, and ESAVI/1068/04.10.07/2017). All birds were captured, handled, and ringed with an appropriate ringing license.

## Results

At the population level, across the years studied, the total offspring sex ratio was slightly male biased with 53.7% (191/366) male.

On a brood level, broods of GxB parents (i.e. G mother × B father) tended to be more female biased than those of BxB parents, and later broods tended to be more male biased than earlier broods (Supplementary Table S1). Laying date had a different effect on brood sex ratio depending on the parent morph combination (Supplementary Table S1). In broods of BxB and BxG parents (i.e. with B mothers), the proportion of sons in the brood increased as the breeding season advanced, whereas in broods of GxG and GxB parents (i.e. with G mothers), the proportion of males decreased over the breeding season. Later in the season, the broods of gray mothers (of GxB and GxG pairs) differed significantly from the reference BxB combination (Supplementary Table S1). No other tested variables or interactions were significant (Supplementary Table S1).

**Fig. 1.**
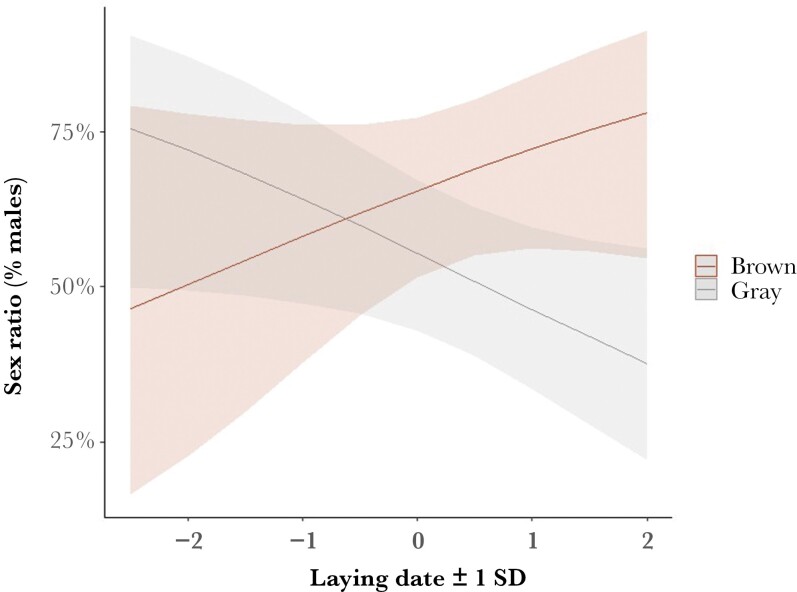
Brood sex ratio (in percentage of males) as a function of (standardized) laying date, grouped by mother morph, as estimated from the model. Shadowed areas indicate 95% CI.

A qualitatively similar model, where the pair morph was replaced with the mother’s morph (Table 1), confirmed the observed pattern. Brown mothers produced female-biased broods early in the season and male-biased broods late in the season, whereas gray mothers followed an opposite seasonal pattern (Fig. 1; Table 1: mother morph by laying date). Also, in this model, mother wing length (a proxy for size) was negatively associated with the proportion of sons in the brood (Table 1). We further constrained our data to only include broods without egg mortality (*N* = 32 broods), i.e. clutch sex ratio. This smaller data set also followed a similar strong pattern with a mother morph by laying date interaction (mother morph by laying date: *z* = 2.55, *P* = 0.011, Supplementary Fig. S1, see Supplementary Table S2 for complete statistics).

**Table 1.** Brood-level sex ratio glmer model output for fixed effects, using brown mothers as reference (*n* = 79). Statistically significant *P* values (*P* < 0.05) in bold.

Fixed effects	Estimate	Std. Error	z	P
(Intercept)	0.421	0.515	0.818	0.414
Mother Morph (Gray)	−0.422	0.264	−1.597	0.110
Mother Mass	0.172	0.135	1.271	0.204
**Mother Wing**	−**0.300**	**0.145**	−**2.075**	**0.038**
Laying date (LD)	0.383	0.250	1.533	0.125
Brood Size	0.001	0.124	0.012	0.991
**Mother Morph by LD**	−**0.666**	**0.297**	−**2.240**	**0.025**

### Offspring condition

**Fig. 2.**
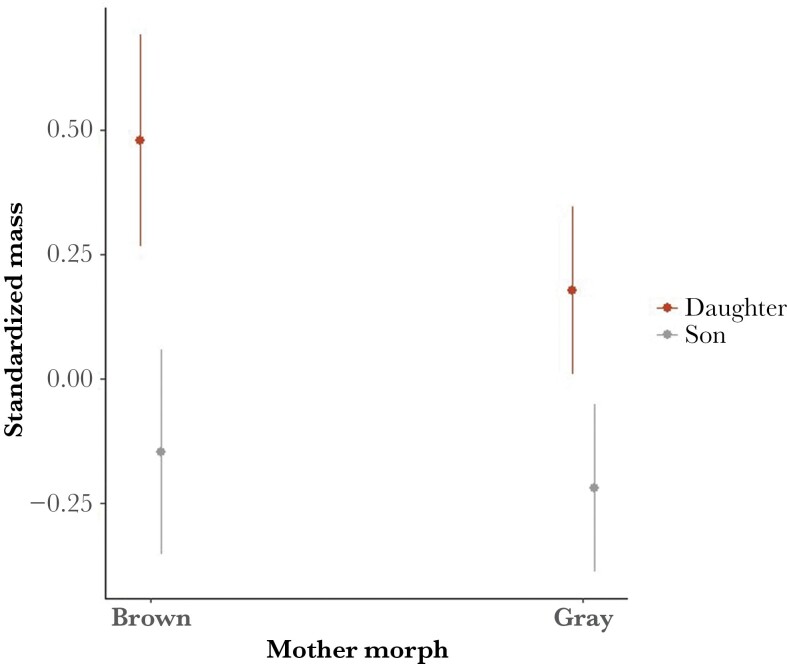
Standardized offspring body mass by offspring sex, based on the mother’s color morph (*n* = 348). Bars represent 95% confidence interval. The figure illustrates the interactive sex-specific effect of mother’s color morph on size-corrected offspring mass (see Table 2 for statistics).

In the “Mother morph model,” offspring of brown mothers were significantly larger than offspring of gray mothers (Table 2). There was a significant interaction of offspring sex × mother morph (Table 2), meaning that the effect of the mother’s morph on offspring mass was dependent on the sex of the offspring. The difference in mass between offspring sexes (size dimorphism) was significantly bigger for offspring of brown mothers than those of gray mothers (Fig. 2).

**Table 2.** Offspring condition lmer model outputs for fixed effects, considering mother morph with gray and female offspring as reference levels (*n* = 348). *P* values for statistically significant results (*P* < 0.05) in bold.

Fixed effects	Estimate	Std. Error	df	t value	Pr(>|t|)
(Intercept)	0.176	0.085	8.847	2.079	0.068
Std. Wing length	0.691	0.031	320.610	22.449	**<0.001**
Sex	−0.394	0.063	279.144	−6.216	**<0.001**
Mother Morph	0.310	0.119	156.446	2.593	**0.010**
Std. laying date	−0.132	0.073	21.744	−1.786	0.088
Mother Morph by Sex	−0.223	0.107	279.609	−2.084	**0.038**
Mother Morph by Std. Laying date	−0.150	0.105	101.091	−1.431	0.156
Sex by Std. Laying Date	−0.006	0.053	288.631	−0.113	0.910

In the alternative “Offspring morph model,” offspring mass was significantly associated with offspring wing length and offspring sex (see Supplementary Table S3; *P* < 0.001 for both variables in all models), such that male offspring were generally smaller than females. Offspring morph alone did not affect offspring mass in this data set (*t* = 0.678, *P* = 0.498, *n* = 348, see Supplementary Table S3 for full statistics). Laying date was negatively associated with offspring condition, such that later-born offspring tended to be smaller than early-born ones (Supplementary Table S3). However, neither the interaction of offspring morph by laying date (Supplementary Table S3) nor offspring sex by laying date (Supplementary Table S3) were statistically significant.

## Discussion

The main results of this study indicate that, while there is no marked deviation from parity in the population sex ratio, there is an effect of timing of breeding, which suggests that females adjust their brood sex ratio plastically to increase the fitness return of their offspring. Additionally, this effect of laying date (which acts as a proxy for food availability) varies depending on the parental morphs, which suggests that genetically determined color morphs vary in their sex allocation strategies according to environmental conditions. More specifically, broods of gray mothers range from mainly male-biased early in the season to female-biased late in the season, whereas broods of brown mothers tend to become more male-biased late in the season. The opposite patterns of sex ratio bias over the course of the season suggest that the fitness returns of sons and daughters (*sensu*Trivers and Willard 1973) differ between color morphs. This result is further supported by evidence that brown offspring are bigger at fledging than gray offspring (Morosinotto et al. 2020). Here, we show that this is especially the case for female brown offspring, which makes them costlier to produce compared to gray and male offspring. Early-laid offspring also tended to be heavier than late-laid offspring, and the heavy brown female offspring appeared to benefit most from being laid early (Morosinotto et al. 2020).

Theories of sex ratio adjustments are based on the larger sex being costlier to produce: unlike most bird species, in raptors, the females are larger and presumably costlier. Instances of sex ratio adjustments in a wide range of bird species with sexual size dimorphism (males larger than females) have been well-documented (Alonso-Alvarez 2006) and are based on differential costs of production for offspring of each sex. While the tawny owl, *Strix aluco*, is already known to display this reverse sexual size dimorphism (Lundberg 1986; Sunde et al. 2003), the analysis of offspring condition in this study provides clear evidence for this dimorphism in the Finnish study population prior to fledging, with male offspring being significantly smaller than females.

While Fisher’s (1930) principle assumption of equal costs of producing males and females guides his frequency-dependent selection theory, there is no evidence of a population-level bias towards the smaller, putatively cheaper sex during the 2009 to 2019 period in our data. We find that offspring sex ratio in the tawny owl varies somewhat around 50% male with an average of 53% male-biased, which is in agreement with previous studies of sex ratio in tawny owls: a weakly male-biased (55%) population-level sex ratio was reported in an earlier study (1999 to 2003) of Finnish tawny owls (Kekkonen et al. 2008), while 50% sex ratio was observed in northern England during 1994 to 1998 (Millon et al. 2010), with no significant annual deviation from parity in either study. However, this population-level analysis alone is not necessarily indicative of a lack of sex ratio adjustments by tawny owl parents. For example, similar quantities of female-biased and male-biased broods in a given year could produce an approximately equal sex ratio at the population level, which highlights the need for further analysis that considers brood and parental traits. Instead, our data appear to follow the assumptions of the Trivers-Willard (Trivers and Willard 1973) hypothesis regarding sex ratio adjustment at the brood level based on phenotypic parental traits. In our analysis, we found evidence that tawny owls adjust their brood sex ratios based on timing of breeding: broods laid later tended to be more male-biased than broods produced earlier in the breeding season. We argue this is likely to be an adjustment of sex ratio at egg-laying and not a consequence of sex-differential mortality in the broods (i.e. sex ratio at fledging) since we found a qualitatively similar and statistically stronger pattern when only including broods without mortality in the model. Our finding also supports the hypothesis that male-biased sex ratios are expected when food conditions are poor (see also Brommer et al. 2003). Timing of breeding, as measured by laying date, is strongly regulated by mammalian prey abundance in the previous year (Korpimäki 1987; Brommer et al. 2002), and this measure accounts for more variation within the study area than prey abundance alone, in terms of both uneven distribution of prey across territories and individual hunting ability. When food resources are limiting, laying tends to occur later; therefore, at the brood level, male-biased sex ratios are also more likely under these conditions. Appleby et al. (1997) found female-biased clutches on UK territories where prey abundance was high. Sasvári and Nushiumi (2005) similarly found that sex ratios were male-biased when parents experienced adverse winter conditions shortly prior to egg laying, where increased ground snow cover reduces successful preying on small mammals and contributes to limited food availability. These previous studies support our results, which themselves provide evidence for sex ratio adjustment in tawny owls, though further investigation and experimental manipulations of nutritional conditions would be necessary to better understand the conditions guiding this sex allocation strategy.

In addition to investigating overall sex ratio adjustments, the main objective of this study was to understand the influence of parental traits (heritable color polymorphism) on this process. If the same genes regulating pheomelanism in tawny owls also regulate genes for physiological processes like hormone expression and function (Ducrest et al. 2008), then different color morphs could have different sex determination processes and thereby different sex ratios. We compared broods produced by different color combinations of parents and found that sex ratio adjustment is indeed dependent on the parents’ color morph. The effect of the laying date was conditional on the mother’s morph, where gray mothers were found to produce more female-biased broods later in the breeding season than brown mothers. On the contrary, brown mothers produce the larger sex (daughters) when they breed early in the season and more males when they breed later. Since there is a seasonal decline in clutch size in northern populations of tawny owls, with larger clutches early in the season (Kekkonen et al. 2008; Morosinotto et al. 2020), it can be concluded from these results that sex ratio adjustment is also influenced by timing of breeding on a phenotypic (color morph) level: as predicted, broods of brown mothers are more strongly male-biased when breeding is delayed to the late season. In another raptor, the common buzzard (*Buteo buteo*), plumage morph of the mother was also found to significantly affect the offspring sex ratio, with intermediate-morph mothers producing more female offspring during periods of low prey abundance than light or dark mothers (Chakarov et al. 2015). These findings suggest that there are inherent differences between the morphs that influence sex allocation strategies.

In the tawny owls and other avian species displaying melanin-based color polymorphism, variation in melanin-based coloration is strongly genetically determined (Cooke et al. 1968; Schmutz and Schmutz 1981; Briggs et al. 2010; Karell et al. 2011; Amar et al. 2013; Kappers et al. 2018), which has allowed investigating how certain color patterns may be associated with variation in physiology and life-history patterns, and provided evidence for pleiotropy as a genetic basis for these associations. A literature review by Ducrest et al. (2008) of the melanocortin system in vertebrates suggests the proopiomelanocortin gene and melanocortin receptor genes are key regulators of melanin-based coloration in vertebrates and also have pleiotropic effects on other physiological and behavioral traits, including endocrinological functions, metabolism, aggression and resistance to stress. Morph-specific physiological traits have been observed in tawny owls, from insulative feather structure (Koskenpato et al. 2016), to responses to food conditions and energy requirements (Piault et al. 2009; Emaresi et al. 2014), offspring mass (Morosinotto et al. 2020), immune challenges (Gasparini et al. 2009; Karell et al. 2011) and telomere dynamics (Karell et al. 2017; Morosinotto et al. 2021, 2022). Here, we find morph-specific differences in sex allocation strategies, which suggests that melanin-based coloration and physiological mechanisms that determine sex allocation may be associated.

While our results support sex ratio adjustment in this species based on the mother’s morph and timing of breeding, it is also important to understand how certain conditions affect offspring of each sex to form the theoretical basis for *why* sex ratio adjustment occurs the way it does in tawny owls. In this study, the offspring morph itself was not found to influence its condition in the late nestling stage, but this result is likely due to the relatively small dataset used here. In a larger study of the same population, brown offspring were consistently heavier than gray offspring prior to fledging (Morosinotto et al. 2020). Additionally, Morosinotto et al. (2020) found that offspring born early were heavier than offspring born later. Experimental cross-fostering studies combined with nutritional manipulation could assess if the observed patterns of offspring size at fledging could be mediated via maternal pre-hatching effects.

Piault et al. (2009) previously showed that the offspring of brown mothers convert food more efficiently to body mass under good (ad lib) food conditions but lost more body mass under poor (restricted) food conditions than those of gray mothers. Offspring of brown mothers could, therefore, be expected to have lower mass than those of gray mothers under poor food conditions. While offspring of brown mothers were found to be significantly heavier than those of gray mothers (Table 2), Morosinotto et al. (2020) additionally showed that this was the case even in later produced broods (pair morph by laying date interaction). Here, we also showed a significant interaction between offspring sex and female morph (Fig. 2), such that daughters of brown mothers were especially heavier than those of gray mothers. The difference in condition between siblings of opposite sexes is also largest in broods of brown mothers (Fig. 2). This suggests that brown mothers would produce more daughters early in the season/under good food conditions. Given that (1) brown offspring are consistently heavier than gray offspring, (2) that this effect is most pronounced in female offspring, and (3) that later laying date is linked to poor food conditions, it can be argued that female offspring benefit most in terms of overall condition when they are born early/under good conditions to brown mothers.

The findings of this study and previous studies discussed here support the theory that sex ratio adjustments in tawny owls may be adaptive. Tawny owl offspring with higher mass at fledgling are more likely to recruit to the breeding population the following year (Morosinotto et al. 2020). Emaresi et al. (2014) had previously predicted that darker (brown) tawny owls favor offspring quality over number and demonstrated that brown parents have relatively constant reproductive effort that is independent of environmental conditions. We showed that daughters of brown mothers are consistently heavier than daughters of gray mothers; thus, the benefits of producing female offspring are highest for brown mothers, especially during the early breeding season, when food is abundant. Although we cannot infer any causal relationships, we propose that it can be adaptive for brown mothers to produce less female offspring later in the breeding season (i.e. in poor food conditions), as observed in this population study, in favor of cheaper males. The body mass difference between sons and daughters is smaller among offspring of gray mothers, which may suggest the fitness returns of sons and daughters would follow a different pattern (offspring number over quality, *sensu*Emaresi et al. 2014) than that of brown mothers. However, our data do not allow an assessment of whether the actualized fitness returns from sons and daughters differ depending on color morph and season, which would be required to confirm our interpretation of the pattern we observe.

Regarding the higher prevalence of female offspring of gray mothers later in the season and under poor food conditions observed in this study, it has been suggested that the offspring of gray mothers outcompete those of brown mothers under harsh conditions (Roulin et al. 2008; Piault et al. 2009). Offspring of gray mothers do not convert food to body mass as efficiently in rich food conditions as those of brown mothers but also do not lose as much in restricted food conditions (Piault et al. 2009). Broods of gray mothers laid earlier may face more competition for food and, therefore, favor less costly male offspring: female nestlings would require more food for the same relative increase in mass compared to their male siblings. Gray parents have also been shown to adjust their reproductive effort relative to environmental conditions (Emaresi et al. 2014), which may mitigate some of the “loss of benefit” when broods are laid later and explain why sex ratios of gray-mother broods do not skew as much as those of brown parents as the season progresses. Overall, these findings suggest that genetically determined phenotypes (morphs) adjust their sex ratio differently according to environmental cues and that such adjustments are likely to improve fitness.

## Supplementary Material

arae039_suppl_Supplementary_Tables_S1-S3_Figure_S1

## Data Availability

Analyses reported in this article can be reproduced using the data provided by Tooth et al. (2024).
