# Supplementary material for: Sex allocation is color morph-specific and associated with fledging condition in a wild bird
Source: Behav Ecol. 2024 May 17;35(4):arae039. doi: 10.1093/beheco/arae039 (PMC11138213; doi:10.1093/beheco/arae039)

Supplementary data for

**Sex allocation is color morph-specific and associated with fledging condition in a wild bird**

**Supplementary table S1**. Brood-level sex ratio glmer model output for fixed effects, using Brown x Brown pairs as the parent (mother x father) morph reference (n=79). Statistically significant *p* values (p < 0.05) in **bold**; non-significant tendencies (0.05 < p < 1) in *italics*.

| **Fixed Effects** | **Estimate** | **Std. Error** | **z value** | **Pr(>\|z\|)** |
| --- | --- | --- | --- | --- |
| *(Intercept)* | 0.703 | 0.648 | 1.085 | 0.278 |
| *Brown x Gray* | -0.226 | 0.523 | -0.432 | 0.666 |
| *Gray x Brown* | -0.935 | 0.512 | -1.825 | *0.068* |
| *Gray x Gray* | -0.420 | 0.507 | -0.827 | 0.408 |
| *Mother mass* | 0.219 | 0.142 | 1.545 | 0.122 |
| *Mother wing* | -0.225 | 0.150 | -1.501 | 0.133 |
| *Laying date* | 1.272 | 0.658 | 1.932 | *0.053* |
| *Brood Size* | -0.009 | 0.131 | -0.072 | 0.942 |
| *Brown x Gray by Laying date* | -1.125 | 0.705 | -1.595 | *0.111* |
| *Gray x Brown by Laying date* | -1.616 | 0.682 | -2.370 | **0.018** |
| *Gray x Gray * Laying date* | -1.586 | 0.706 | -2.245 | **0.025** |

**Supplementary table S2.** Brood-level sex ratio glmer model output for fixed effects, using Brown mothers as reference and considering only broods with no egg or nestling mortality (n=32). Statistically significant *p* values (p < 0.05) in **bold**.

| **Fixed effects** | **Estimate** | **Std. Error** | **z** | **p** |
| --- | --- | --- | --- | --- |
| *(Intercept)* | 2.235 | 1.16 | 1.932 | *0.053* |
| *Mother Morph (Gray)* | -0.700 | 0.392 | 1.787 | 0.074 |
| *Laying Date (LD)* | 0.104 | 0.280 | 0.370 | 0.711 |
| *Brood Size* | -0.344 | 0.26 | -1.319 | 0.187 |
| ***Mother Morph by LD*** | -1.025 | 0.402 | 2.548 | **0.011** |

**Supplementary table S3.** Offspring condition lmer model outputs for fixed effects, considering offspring morph with gray and female offspring as reference levels (n=348). P values for statistically significant results (p < 0.05) in **bold**. “Std.” indicates standardized variables; see Methods above for detailed explanations of variables and standardization.

| **Fixed effects** | **Estimate** | **Std. Error** | **df** | **t value** | **Pr(>\|t\|)** |
| --- | --- | --- | --- | --- | --- |
| *(Intercept)* | 0.255 | 0.082 | 7.152 | 3.117 | **0.017** |
| *Wing length* | 0.691 | 0.031 | 317.103 | 22.182 | **< 0.001** |
| *Sex* | -0.432 | 0.066 | 270.374 | -6.538 | **< 0.001** |
| *Color Morph* | 0.058 | 0.086 | 326.680 | 0.678 | 0.498 |
| *Laying date* | -0.199 | 0.075 | 18.717 | -2.670 | **0.015** |
| *Color Morph by Sex* | -0.113 | 0.104 | 278.851 | -1.089 | 0.277 |
| *Color Morph by Laying date* | 0.060 | 0.067 | 338.689 | 0.903 | 0.367 |
| *Sex by Laying Date* | -0.017 | 0.053 | 289.577 | -0.319 | 0.750 |

**Supplementary figure S1.** Sex ratio (in percentage of males) of broods at egg-laying as a function of (standardized) laying date, grouped by mother morph, as estimated from the model. Data include only broods where no mortality occurred between egg-laying and fledging. Shadowed areas indicate 95% CI.


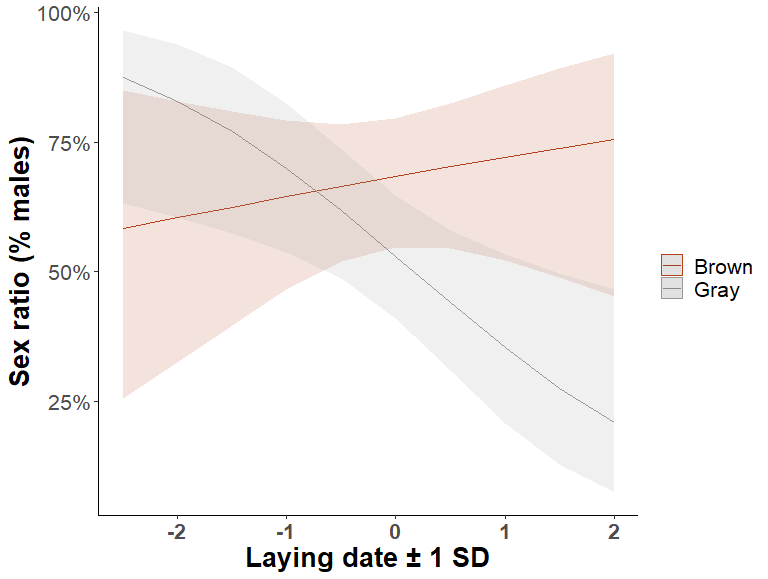

Supplement: arae039_suppl_Supplementary_Tables_S1-S3_Figure_S1 [file arae039_suppl_supplementary_tables_s1-s3_figure_s1.docx]
